# Using a random forest model to predict volume growth of larch, birch, and their mixed forests in northern China

**DOI:** 10.3389/fpls.2025.1682940

**Published:** 2025-12-02

**Authors:** Junfei Zhang, Ziyi Wang, Minghao Li, Xiaotong Chen, Yue Pang, Zhidong Zhang

**Affiliations:** College of Forestry, Hebei Agricultural University, Baoding, Hebei, China

**Keywords:** carbon neutrality, random forest algorithm, stand volume growth, relative importance, partial dependence

## Abstract

Accurately quantifying forest volume and identifying its driving mechanisms are critical for achieving carbon neutrality objectives. Using data from the National Forest Inventory (NFI), plot-level measurements, and environmental variables from pure larch (LP), birch (BP), and mixed larch-birch (LB) forests in the mountainous region of northern Hebei, China, this study employed random forest (RF) algorithms to evaluate the relative importance and partial dependence of biotic and abiotic factors on stand volume growth. A total of 33 predictors related to climate, topography, and soil were analyzed, and model hyperparameters were optimized through grid search combined with blocked cross-validation to mitigate spatial autocorrelation. The RF models exhibited strong predictive performance, with the BP model achieving the highest *R*² (0.92). The minimum temperature of the coldest month (Bio12) was identified as the most influential predictor across all stand types, while stand age also exerted a substantial effect on growth dynamics. Young and middle-aged forests demonstrated higher productivity compared with near-mature and mature stands, suggesting that the latter require improved management interventions to sustain growth. The LB stands exhibited higher productivity than pure stands, likely due to species complementarity and interspecific facilitation. In LP, growth was primarily driven by the interaction between stand age and canopy density, whereas in BP, slope position was more decisive. The management of LB stands offers potential to maintain or enhance forest productivity. The findings emphasize the importance of adaptive forest management strategies that optimize forest structure and mitigate climate change impacts. These insights contribute to advancing carbon sequestration efforts and supporting the development of carbon neutrality policies by enhancing forest productivity and resilience to climate variability.

## 1 Introduction

Global climate change, primarily driven by anthropogenic activities, poses a profound threat to ecosystems and human well-being (Malhi et al., 2002; Solomon et al., 2009; Rehman et al., 2021). In response, the Chinese government has established the ambitious “dual carbon” goals, aiming to peak CO_2_ emissions by 2030 and achieve carbon neutrality by 2060. Enhancing forest carbon sequestration has been recognized as a key national strategy for achieving these goals, as forests constitute the largest terrestrial carbon pool (Pan et al., 2011). Forest stocking volume, serving as a direct proxy for biomass and carbon storage, represents a critical metric for assessing carbon sink capacity (Aalde et al., 2006). The National Forestry and Grassland Administration has set explicit targets to increase national forest coverage to 24.1% and forest volume stock to 19 billion m³ by 2025 (National Forestry and Grassland Administration, 2021). Therefore, accurately quantifying forest volume and identifying its driving mechanisms are essential for developing scientifically informed forest management practices and supporting climate change mitigation efforts.

Forest volume growth is governed by the complex interplay among stand attributes, climatic conditions, soil properties, and topographic factors (Lei, 2019). Traditional statistical models have been widely used to examine these relationships; however, they often fail to adequately capture the inherent nonlinearity and intricate interactions among multiple driving factors (Tian et al., 2022). This limitation underscores the need for advanced analytical approaches capable of handling such ecological complexity. In recent years, machine learning (ML) techniques, particularly the Random Forest (RF) algorithm, have emerged as powerful tools in ecological research due to their high predictive accuracy and ability to model nonlinear patterns without requiring predefined functional relationships (Breiman, 2001). The RF approach has proven effective in various forest modeling applications, including the estimation of basal area increment (Jevšenak and Skudnik, 2021) and the identification of key biomass drivers (He et al., 2022), demonstrating strong potential for elucidating the combined effects of site and climatic factors on forest growth (Tian et al., 2022).

Larch (*Larix principis-rupprechtii*) and birch (*Betula platyphylla*) forests are ecologically and economically vital in northern China. Previous studies have primarily focused on individual aspects of their productivity, including climate-growth relationships (Wang et al., 2022a; Liu et al., 2024), the impact of stand age and site conditions on productivity (Zhao et al., 2015; Dincă et al., 2020), and the effects of soil quality (Wang et al., 1998; Zhang et al., 2023). Although some studies have reported facilitative effects in mixed stands (Zhang et al., 2022b), a comprehensive assessment that integrates multiple ecological drivers such as stand characteristics, climate, soil, and topography to accurately model volume growth across pure and mixed stands remains lacking. This knowledge gap hinders our ability to predict forest dynamics under changing environmental conditions and to optimize management strategies aimed at enhancing carbon sequestration.

To address these research gaps, this study seeks to answer the following scientific questions: (1) How do the key drivers of volume growth and their relative importance differ among the three stand types? (2) Do mixed stands exhibit higher productivity than pure stands, and what factors contribute to this potential advantage? (3) What are the critical thresholds and nonlinear responses of volume growth to major environmental gradients? Accordingly, the specific objectives were to: (1) develop volume growth models for the three stand types using RF; (2) quantify the relative importance of predictors on volume growth; and (3) explore the response patterns of key variables influencing stand volume growth. The findings are expected to provide a scientific basis for precision forestry management and contribute to regional carbon neutrality initiatives.

## 2 Materials and methods

### 2.1 Data collection and processing

#### 2.1.1 Study area and stand volume growth data

**Figure 1:**
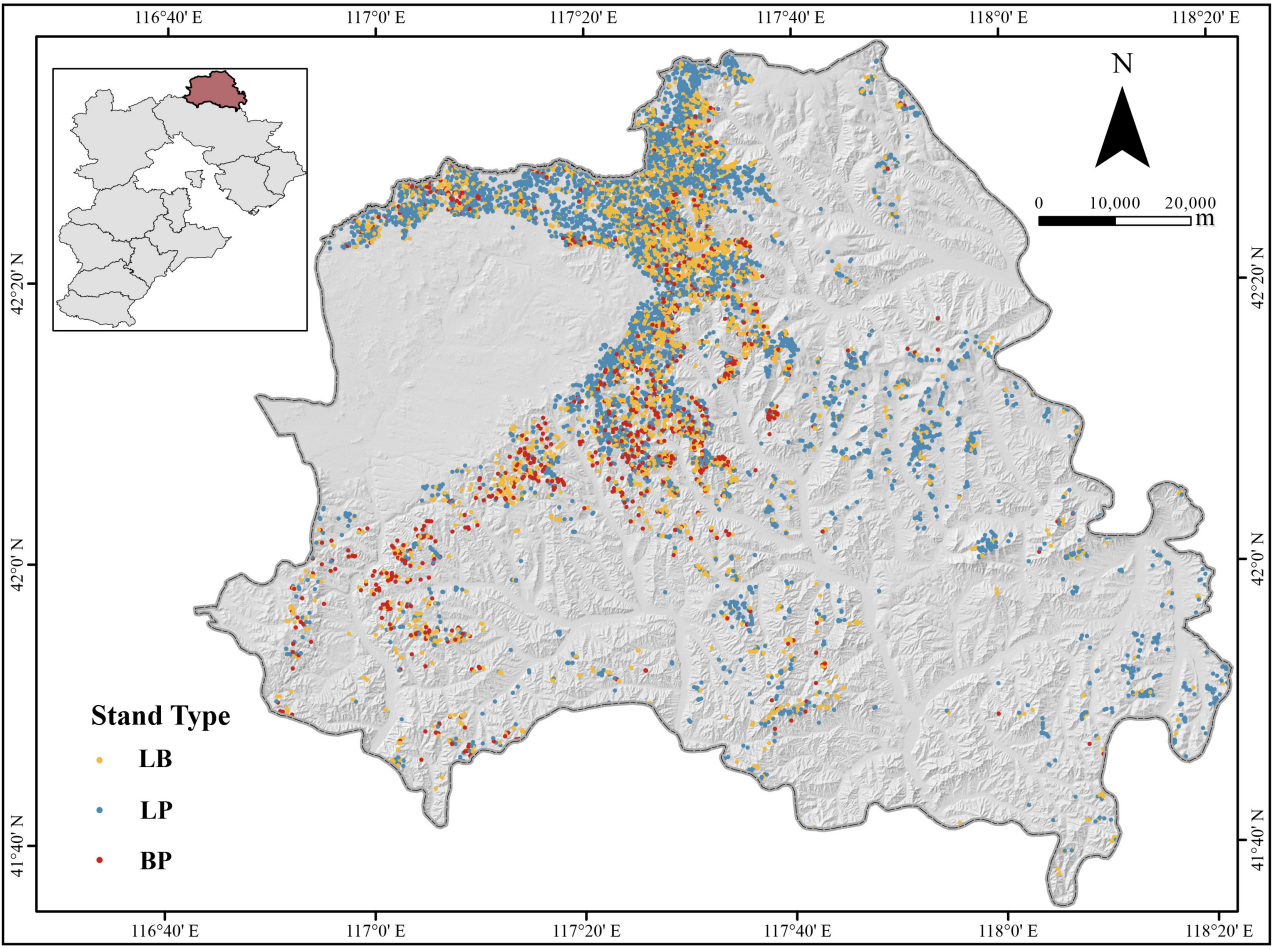
Geographical location of the study area and distribution of sample plots. LP, *Larix principis-rupprechtii* pure forest; BP, *Betula platyphylla* pure forest; LB, mixed *L. principis-rupprechtii–B. platyphylla* forest.

This study was conducted in Weichang County (42°02′–42°36′N, 116°51′–117°39′E), Chengde City, Hebei Province, located at the ecotone between the North Hebei Mountains and the Mongolian Plateau (**Figure 1**). The area encompasses two forest farms: Saihanba Mechanical Forest Farm and Mulan Weichang National Forest Farm. According to the Ninth National Forest Inventory, the primary forest types in the study area were larch plantation, covering 38,182 ha (51.1% of the total forest area), followed by birch secondary forest, which occupies 16,039 ha (21.4%). Larch stands exhibited the highest unit volume at 172 m³/ha, while birch stand averaged 124 m³/ha (Zhang et al., 2025). Stand volume data were derived from three sources. First, subcompartment inventory data (2016 and 2022) from both forest farms were used, encompassing 8,996 subcompartments dominated by larch or birch. The volume for each subcompartment was calculated as the sum of individual tree volumes, with volume growth derived from the difference between 2022 and 2016 measurements. Second, data were extracted from the eighth and nineth forest resource inventories of Hebei Province, which included 123 sample plots in larch and birch stands, typically set up as squares of 0.0667 ha. Third, field survey data (2016 and 2022) from 128 sample plots—set as rectangular plots ranging from 600 to 2,500 m²—were incorporated. Individual tree volume was calculated using two-variable standing volume models (Equations 1, 2) based on diameter at breast height (DBH) and tree height. Stand volume was calculated as the total volume of all trees per plot, while volume increment was computed as the difference between the two survey years. Notably, only alive trees that exist in both periods were counted. A stand was classified as pure larch or pure birch if the dominant species accounted for more than 80% of the total stand volume; otherwise, it was designated as mixed larch-birch stand. To ensure comparability, the volume increment was standardized to an annual per-hectare rate (m³·ha^-^¹·yr^-^¹), adjusted based on plot area and the duration of the growth period.

Equations 1, 2 were used to estimate the individual tree volume for larch and birch, respectively (Standardization Administration of the People’s Republic of China, 2024).

(1) $$V=0.00007267D^{1.80035}\times H^{0.99246}$$

(2) $$V=0.00008077D^{1.83864}\times H^{0.89108}$$

where: *V* is the standing tree volume (m^3^); *D* is the diameter at breast height (DBH, cm); and *H* is the tree height (m). The single-tree volume equations were calibrated based on data collected from a broad region of northern China, with the detailed parameters of each equation presented in **Supplementary Table S2** of the **Supplementary Material**.

#### 2.1.2 Bioclimatic factors

Bioclimatic data were obtained using ClimateAP software (v3.10) (http://ClimateAP.net). ClimateAP provides location-specific monthly and seasonal climate variables across the Asia-Pacific region for the period 1901–2100 (Wang et al., 2017b). For each plot’s specific growth period, a corresponding time series of annual climate data was obtained. The annual values of each climatic variable were then averaged over the growth period, resulting in a dataset comprising 15 time-averaged bioclimatic variables for each sample plot (**Table 1**).

**Table 1:** Candidate variables for stand volume growth modeling.

Type	Variable	Mean	SD	Min.	Max.
Stand attributes	Δ*V* (m³·ha^-^¹·yr^-^¹)	10.2	6.1	1.7	27.9
	Stand age (yr)	39.2	10.2	10.0	90.0
	Canopy density	0.7	0.2	0.1	0.9
Topographic factors	Altitude (m)	1530.0	185.5	668.0	2034.0
	Slope (°)	11.8	7.2	0.0	40.7
	Slope direction	–	–	–	–
	Slope position	–	–	–	–
Soil factors	AK (mg·kg^-1^)	173.5	56.0	43.1	308.3
	AN (mg·kg^-1^)	157.2	62.8	13.2	501.0
	AP (mg·kg^-1^)	10.6	5.1	1.9	16.3
	TK (mg·kg^-1^)	1789.0	375.5	1178.0	2485.0
	TN (mg·kg^-1^)	255.5	100.8	10.9	498.6
	TP (mg·kg^-1^)	71.9	15.7	30.8	112.4
	BD (g·cm^-3^)	1.3	0.1	0.8	1.4
	CL (%)	16.2	1.3	5.1	20.9
	GRAV (%)	15.8	7.4	0.0	42.0
	SA (%)	41.2	3.3	29.8	82.8
	SOM (%)	4.7	2.1	0.2	10.7
	pH	6.7	0.4	6.0	8.5
Climatic factors	Bio1 (°C)	2.3	1.2	0.2	8.5
	Bio2 (°C)	18.9	0.9	16.8	23.5
	Bio3 (°C)	-16.9	1.3	-19.4	-9.4
	Bio4 (°C)	35.7	0.5	30.7	37.8
	Bio5 (mm)	516.3	25.2	382.5	749.2
	Bio6 (days)	151.6	12.1	128.0	203.2
	Bio7 (°C)	17.0	1.0	14.7	22.3
	Bio8 (°C)	-14.3	1.3	-16.7	-6.9
	Bio9 (mm)	328.3	17.4	222.5	494.2
	Bio10 (mm)	17.4	5.3	6.3	26.5
	Bio11 (°C)	25.1	0.8	23.3	29.0
	Bio12 (°C)	-20.4	5.0	-26.0	-10.3
	Bio13 (°C)	13.9	0.5	11.6	15.2
	Bio14 (mm)	158.3	9.1	97.5	235.5
	Bio15 (mm)	3.0	2.3	0.9	40.8

(1) “-” represents categorical variables; SD, standard deviation; Min, minimum; Max, maximum. (2) Δ*V*, average annual volume growth of forest stand; Stand age, average age of dominant tree species; Canopy density, the ratio of the tree canopy cover to the total forest area; AK, available K; AN, alkali-hydrolysable N; AP, available P;TK, total K; TN, total N; TP, total P; BD, bulk density; CL, percentage of clay; GRAV, gravel; SA, percentage of sand; SOM, soil organic matter; pH, soil pH value in the topsoil; Bio1, annual mean temperature; Bio2, mean warmest month temperature; Bio3, mean coldest month temperature; Bio4, monthly temperature difference (temperature difference between Bio2 and Bio3); Bio5, annual precipitation; Bio6, the number of frost-free days; Bio7, mean temperature of summer; Bio8, mean temperature of winter; Bio9, summer precipitation; Bio10, winter precipitation; Bio11, max temperature of warmest month; Bio12, min temperature of coldest month; Bio13, mean diurnal range [mean of monthly (max temp. – min temp.)]; Bio14, wettest-month precipitation; Bio15, driest-month precipitation.

#### 2.1.3 Topographic factors

Topographic variables—including elevation, slope, aspect, and slope position—were derived from a 30-m resolution Digital Elevation Model (DEM) obtained from the National Earth System Science Data Center (http://www.geodata.cn). Elevation, slope, and aspect were extracted from the DEM using ArcGIS 10.8, while slope position was recorded for each sample plot during field surveys. All DEM-based layers were projected onto the CGCS2000/Gauss–Krüger Zone 20 coordinate system to ensure spatial consistency. Raster alignment was subsequently checked to confirm that all layers shared the same extent, grid origin, and pixel alignment.

#### 2.1.4 Soil factors

Soil data were sourced from the National Earth System Science Data Center (http://www.geodata.cn). Twelve soil variables were selected for analysis (**Table 1**). The original soil data, with a spatial resolution of 900 m, were resampled to 30 m using bilinear interpolation in ArcGIS 10.8 to ensure consistency with other variables. All soil layers were projected onto the CGCS2000/Gauss–Krüger Zone 20 coordinate system, and raster alignment was checked to ensure consistent extent, grid origin, and pixel alignment across all spatial layers.

### 2.2 Forest type classification

Landsat 8 OLI Level-2 surface reflectance imagery was obtained from the Geospatial Data Cloud (http://www.gscloud.cn) at a spatial resolution of 30 m. As July was identified as the period during which chlorophyll content peaked in the canopies of both larch and birch (Asner and Martin, 2008), the acquisition date was set to July 24, 2022. The near-infrared band (Band 5, 0.85–0.88 μm) and the shortwave infrared band (Band 7, 2.11–2.29 μm), which are sensitive to spectral differences between coniferous and broadleaf species, were used as key inputs for supervised classification (Liu et al., 2018; Soleimannejad et al., 2019).

Supervised classification of forest types was performed using the Random Forest (RF) algorithm in ArcMap 10.8, based on satellite imagery and plot data (see **Supplementary Figure S1** for classification results). Training and validation samples were derived from field survey data. A stratified random sampling strategy was employed to select these samples, effectively mitigating spatial dependence by ensuring geographic separation between the training and validation datasets. A confusion matrix was then constructed to evaluate classification accuracy. Four accuracy metrics, including Producer’s Accuracy (PA), User’s Accuracy (UA), Overall Accuracy, and the Kappa coefficient (*K*) (Olofsson et al., 2014; Morales-Barquero et al., 2019; Xie et al., 2019; Olorunfemi et al., 2020), were calculated. The classification yielded an Overall Accuracy of 91.11% and a *K* of 86.67%, with detailed results provided in **Supplementary Table S1** of the **Supplementary Material**.

### 2.3 Random forest algorithm

RF is an ensemble learning method that combines multiple models to enhance overall predictive performance. An RF model comprises several decision trees and operates based on three core steps. First, *n* samples (where *n* equals the size of the original dataset) are randomly drawn with replacement from the original dataset to form a training subset. Second, a regression tree is fitted to each subset. At each node split, a random subset of features is selected, and the optimal split is determined based on variance reduction. This recursive process continues until terminal nodes contain either samples from a single class or no more than five observations. Finally, predictions from all individual trees are averaged to produce the final output (Breiman, 2001; Ou et al., 2019).

Forest ecosystems are characterized by multi-source, high-dimensional data involving topography, climate, and stand structure. The powerful nonlinear modeling capability of RF allows for the complex coupling among these factors. In previous studies on forest productivity, RF models have consistently demonstrated superior performance compared to alternative approaches (Hamidi et al., 2021; Liu et al., 2022; Zhang et al., 2022a). However, the internal decision-making logic of RF is difficult to interpret intuitively, and the relationship between input features and model output remains opaque. To enhance interpretability, relative importance analysis and Partial Dependence Plots (PDPs) were employed (Breiman, 2001; Friedman, 2001). This combined approach has been widely adopted in ecological modeling (Jevšenak and Skudnik, 2021; Wang et al., 2021; He et al., 2022; Tian et al., 2022; Wang et al., 2022b; Zhang et al., 2022a).

For regression task, the relative importance of each predictor variable was determined using the Percent Increase in Mean Squared Error (%IncMSE) metric (Genuer et al., 2012; Kuhn, 2008). The resulting importance values were normalized as percentages of the total predictor importance for comparative analysis (Tian et al., 2022). Based on these rankings, PDPs were generated for the top eight predictors influencing volume growth, using the Locally Weighted Regression (LOESS) smoothing method (Cleveland and Devlin, 1988; Jacoby, 2000), which effectively reduces high-frequency noise while preserving critical ecological thresholds (Shi et al., 2019; Wang, 2024). To further improve model interpretability, the interaction strength among variables was quantified using Friedman’s H-statistic (Friedman and Popescu, 2008), and the marginal effects of the top eight predictors were visualized through Accumulated Local Effects (ALE) plots, which account for potential feature correlations (Apley and Zhu, 2020). Additionally, SHapley Additive exPlanations (SHAP) values were calculated to assess the contribution of each predictor to individual model outputs (Lundberg et al., 2020). All interpretability analyses were performed using the “iml” package (Molnar et al., 2018), and the RF model was implemented using the “randomForest” package (Genuer et al., 2012) in R version 4.3.2.

### 2.4 Model development

A total of 33 predictor variables (**Table 1**) were selected as independent variables to model stand volume growth using the RF algorithm. During RF model construction, three key hyperparameters required tuning: the number of regression trees (*ntree*), the number of predictor variables randomly selected at each tree node for splitting (*mtry*) (Tian et al., 2022), and the minimum node size (*min.node.size*). Generally, the overall error rate decreases with increasing *ntree* and stabilizes once *ntree* exceeds 500. To ensure model reliability while maintaining computational efficiency, *ntree* was set to 1000 in this study (**Supplementary Figure S2**) (Zhang et al., 2016). Althought *mtry* was often set to one-third of the total number of predictors in previous studies (Breiman, 2001), the optimal *mtry* value was typically variable. Therefore, a comprehensive grid search was performed over the full range of *mtry* values (1–33) and five *min.node.size* values (3, 5, 10, 15, and 20). Hyperparameter tuning was carried out using the “caret” package (Kuhn, 2008) with the “ranger” method in R. Model selection followed the one-standard-error (one-SE) criterion, which selects the most parsimonious model whose cross-validation error falls within one standard error of the minimum, thereby improving generalizability and model stability.

To account for differences in tree species composition, all samples were categorized into three forest types: pure larch (LP), pure birch (BP), and mixed larch–birch (LB) forests. For each forest type, spatial clustering was first applied to partition the samples into 10 spatially coherent clusters. These clusters were then used to divide the data into training and testing sets at an 8:2 ratio, ensuring that the residuals of the testing set exhibited no significant spatial autocorrelation. Independent RF models were then developed for each forest type using their respective training datasets.

### 2.5 Model evaluation

Traditional random or stratified *k*-fold cross-validation can produce overly optimistic performance estimates when applied to spatial data, as nearby samples often exhibit spatial autocorrelation. To address this issue, a blocked cross-validation approach that explicitly accounts for spatial dependence was adopted (Roberts et al., 2017). Specifically, the training samples for each forest type were divided into 10 spatial clusters based on geographic coordinates. A 10-fold blocked cross-validation was then performed, in which each cluster was used once as a validation set while the remaining nine clusters served as the training data (**Supplementary Figures S3-S5**). This procedure effectively reduces spatial leakage and provides a more realistic assessment of model predictive performance.

Model evaluation metrics included the coefficient of determination (*R*²), root mean squared error (RMSE), and mean absolute error (MAE) (**Equations 3**–**5**). The optimal hyperparameter combination was determined based on the one-SE criterion described above. Finally, the predictive accuracy of each optimized RF model was independently validated using the reserved test set to ensure unbiased assessment of model performance.

Cross-validated coefficient of determination ( $R_{\text{CV}}^{2}$):

(3) $$R_{CV}^{2}=\frac{1}{k}\sum_{j=1}^{k}(R_{CV}^{2})=\frac{1}{k}\sum_{j=1}^{k}(1-\frac{\sum_{i=1}^{n_{j}}{\left(\text{Δ}V_{ij}-\text{Δ}{\hat{V}}_{ij}\right)}^{2}}{\sum_{i=1}^{n_{j}}{\left(\text{Δ}V_{ij}-\text{Δ}{\bar{V}}_{ij}\right)}^{2}})$$

Cross-validated root mean squared error (RMSE_cv_):

(4) $$\begin{array}{c} {\text{RMSE}}_{cv}=\frac{1}{k}\sum_{j=1}^{k}({\text{RMSE}}_{j})\\ =\frac{1}{k}\sum_{j=1}^{k}(\sqrt{\frac{j}{n_{j}}\sum_{i=1}^{n_{j}}{\left(\text{Δ}V_{ij}-\text{Δ}{\hat{V}}_{ij}\right)}^{2}}) \end{array}$$

Cross-validated mean absolute error (MAEcv):

(5) $${\text{MAE}}_{cv}=\frac{1}{k}\sum_{j=1}^{k}({\text{MAE}}_{j})=\frac{1}{k}\sum_{j=1}^{k}(\frac{1}{n_{j}}\sum_{i=1}^{n_{j}}|\text{Δ}{\hat{V}}_{ij}-\text{Δ}V_{ij}|)$$

where, *k* is the number of folds (here, *k* = 10); Δ*V_ij_* is the observed volume growth for the *i*-th sample in the *j*-th fold; $\text{Δ}{\hat{V}}_{ij}$ is the corresponding predicted value; $\text{Δ}{\hat{V}}_{ij}$ is the mean of the observed value in fold *j*; and *n_j_* is the number of samples in the *j*-th fold.The metrics *R_j_*^2^, RMSE*_j_* and MAE*j* represent the evaluation results for the *j*-th fold. $R_{\text{CV}}^{2}$ reflects the model’s goodness-of-fit, with values closer to 1 indicating stronger performance. RMSE_cv_ and MAE_cv_ quantify the prediction error, with lower values signifying better predictive accuracy.

### 2.6 Spatial autocorrelation analysis

To characterize the spatial distribution and clustering patterns of stand volume growth across different forest types, a hotspot analysis (Getis-Ord G_i_^*^) was conducted for pure larch (LP), birch (BP), and mixed larch-birch (LB) forest plots. The Getis-Ord G_i_^*^ statistic identifies local spatial autocorrelation by quantifying the degree to which high or low values are spatially clustered in the study area (Getis and Ord, 1992). The hotspot analysis was performed in ArcGIS 10.8. Stand volume growth was subsequently categorized using the equal-interval classification method to visualize spatial clustering patterns for each forest type.

## 3 Results

### 3.1 Spatial distribution pattern of stand volume growth

**Figure 2:**
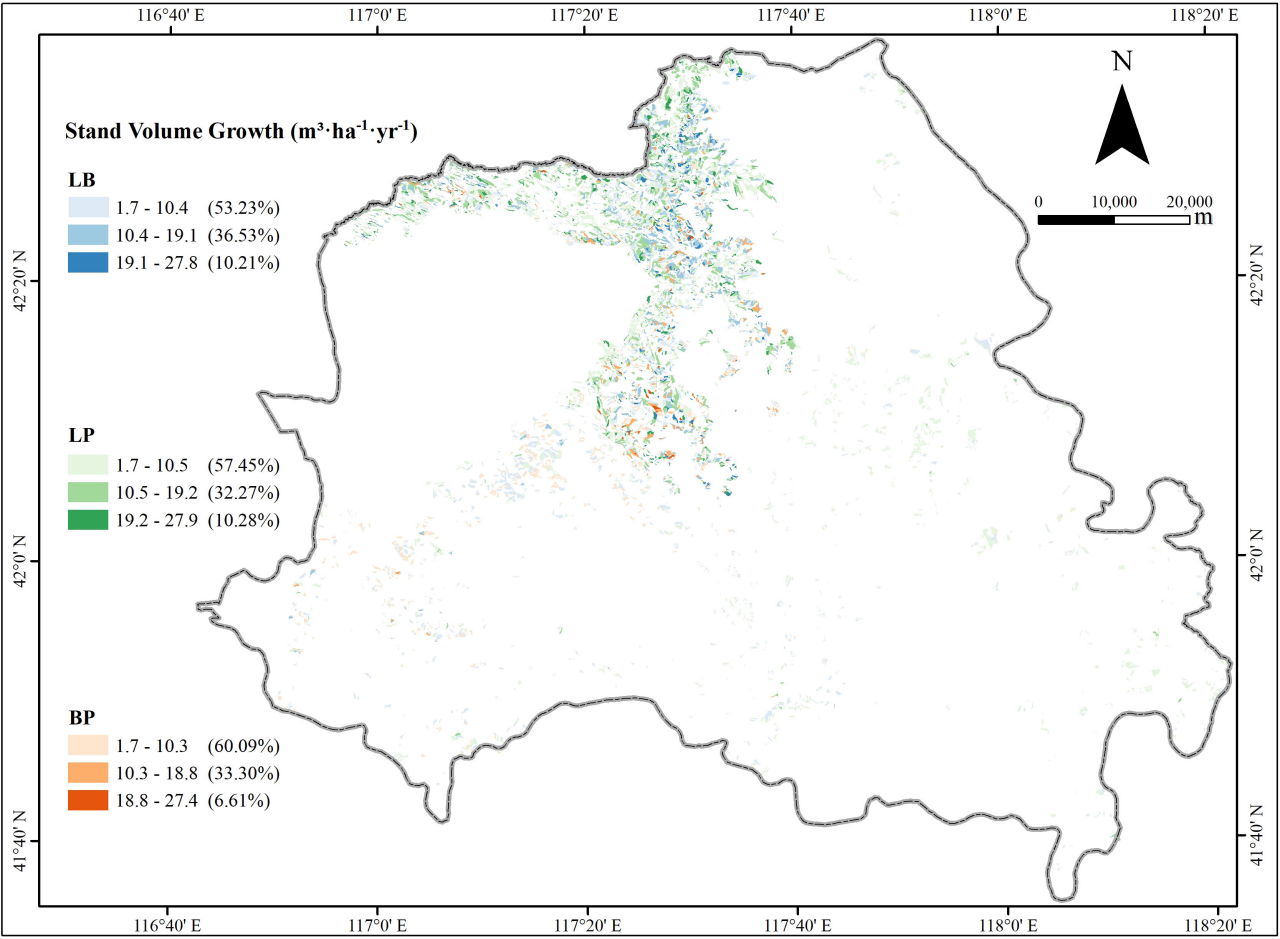
The spatial distribution of the volume growth for the three stand types.

The volume growth of the three forest types (pure larch, pure birch, and mixed larch-birch forest) was categorized into three levels: low, medium, and high, using the equal interval classification method (**Figure 2**). The spatial distribution of volume growth exhibited a similar pattern across all forest types, with low-growth areas dominating the landscape and accounting for more than 50% of the total areas. Hotspot analysis using the Getis-Ord G_i_^*^ statistic revealed that high-growth areas were clustered in the northwestern part of study area, while low-growth areas were concentrated in the southeastern regions (**Supplementary Figure S6**). These findings highlight distinct spatial patterns of stand volume growth, with pronounced high-value clusters in the northwest and low-value clusters in the southeast.

### 3.2 Performance of RF models for stand volume growth

The 10-fold cross-validation procedure yielded three optimized RF models for stand volume growth corresponding to the three forest types. The optimal hyperparameter values were as follows: *mtry* = 5 and *min.node.size* = 15 for LP, *mtry* = 8 and *min.node.size* = 5 for BP, and *mtry* = 8 and *min.node.size* = 10 for LB. Among the models, the BP model achieved the highest predictive performance, with *R*^2^ = 0.92, RMSE = 1.89, and MAE = 1.24. The LB model followed, with *R*^2^ = 0.88, RMSE = 2.68, and MAE = 1.89, while the LP model showed the lowest performance, with *R*^2^ = 0.81, RMSE = 3.28, and MAE = 2.31.

**Figure 3:**
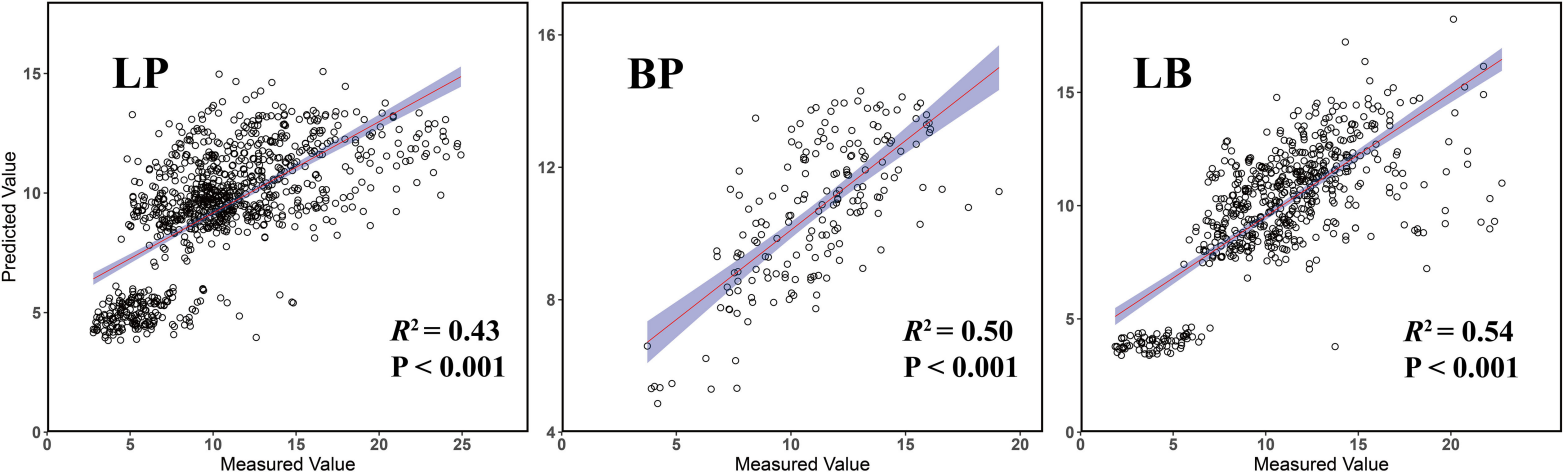
Validated prediction of random forest regression models of three forest types.

Model performance was further evaluated using an independent test set (**Figure 3**). The LB model achieved the highest predictive accuracy, with an *R*^2^ of 0.54, RMSE of 2.73, and MAE of 1.91. The BP model followed, with an *R*^2^ of 0.50, RMSE of 2.05, and MAE of 1.64, while the LP model showed the lowest performance, with an *R*^2^ of 0.43, RMSE of 3.53, and MAE of 2.49. Additionally, residual analyses of the test set revealed no significant spatial autocorrelation across all three models (**Supplementary Figure S7**), indicating that the predictions were not influenced by spatial dependencies and confirming the models’ robustness and generalizability.

### 3.3 Relative importance of variables

**Figure 4:**
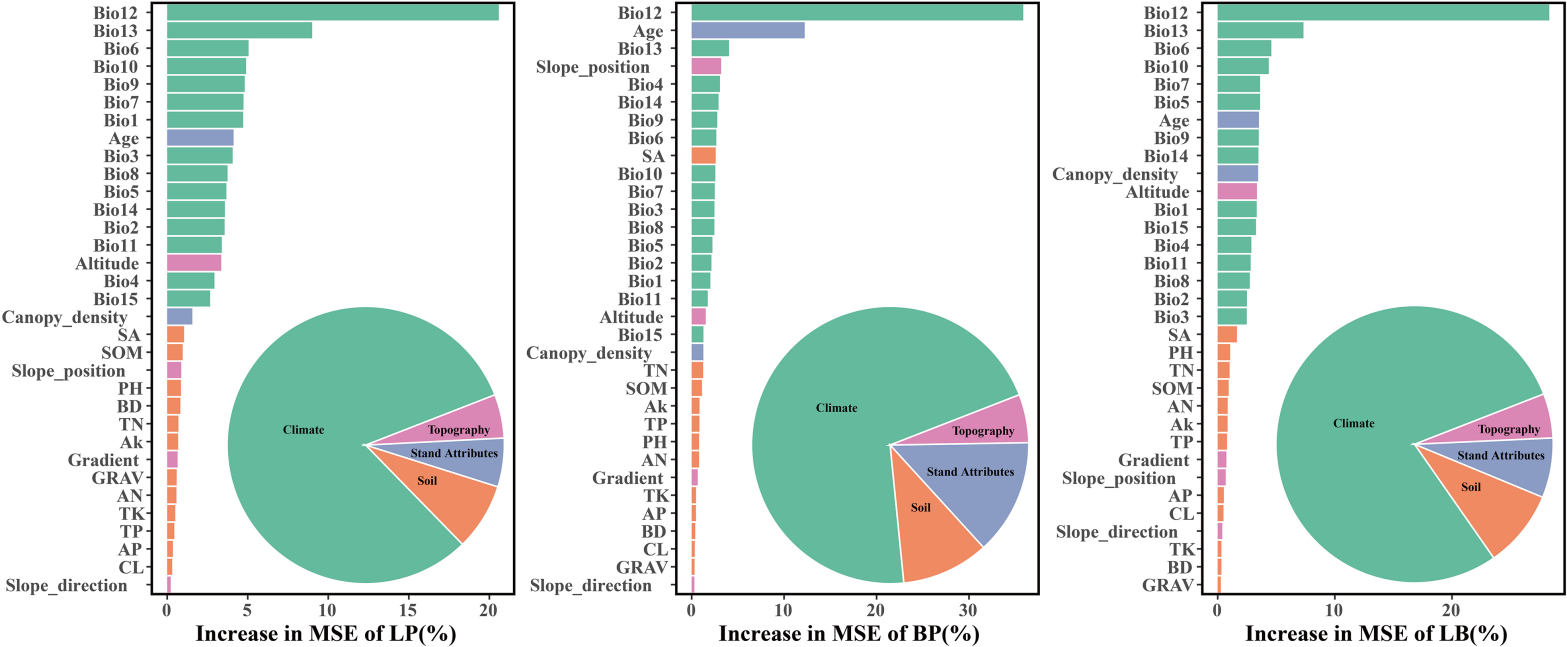
Relative importance of each variable.

**Figure 5:**
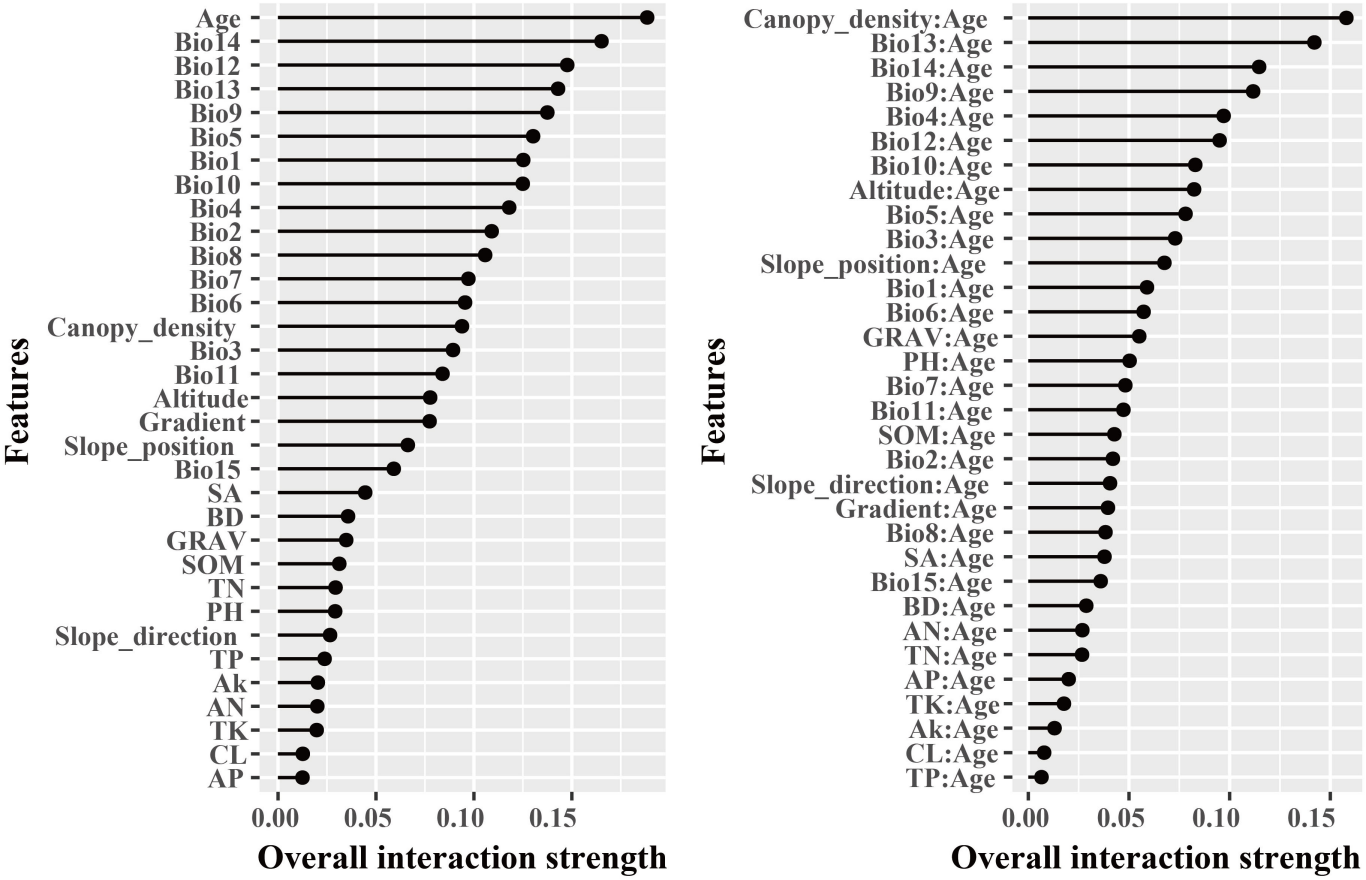
Friedman’s H-statistic interaction for LP.

**Figure 6:**
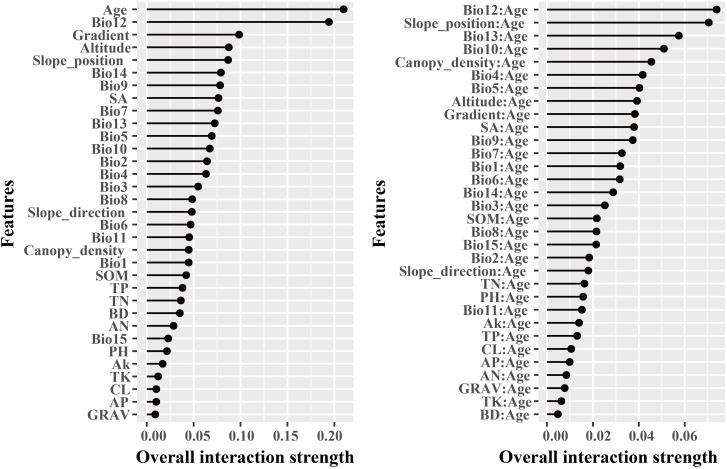
Friedman’s H-statistic interaction for BP.

**Figure 7:**
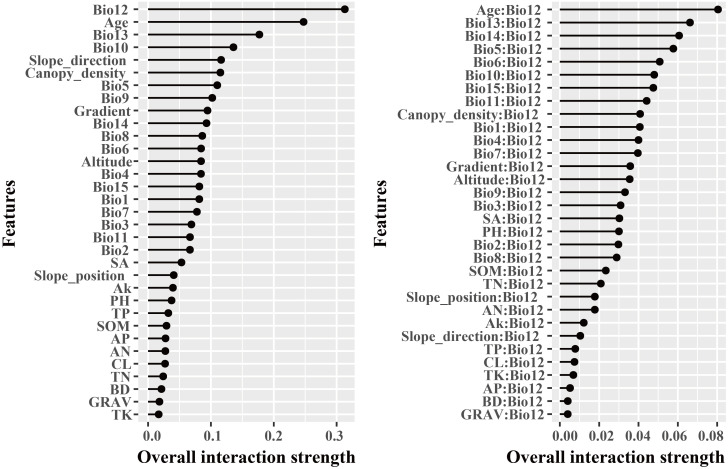
Friedman’s H-statistic interaction for LB.

The dominant factors influencing stand volume growth were generally consistent across the three forest types, with the minimum temperature of the coldest month (Bio12) identified as the most influential predictor in all models (**Figure 4**). Specifically, Bio12 contributed 20.6%, 35.9%, and 28.3% to the total relative importance in the LP, BP, and LB models, respectively. Friedman’s H-statistic further revealed that stand age exhibited the strongest overall interaction effects in the LP and BP models, with H values of approximately 0.2 in both cases (**Figures 5**, **6**). In the LP model, the most pronounced interaction occurred between stand age and canopy density (*H* = 0.16), whereas in the BP model, the interaction between stand age and Bio12 was dominant (*H* = 0.07). In contrast, the LB model showed that Bio12 had the highest overall interaction strength (*H* = 0.31, **Figure 7**), with its strongest interaction observed with stand age (*H* = 0.08).

### 3.4 Partial dependence relationships of predictors

**Figure 8:**
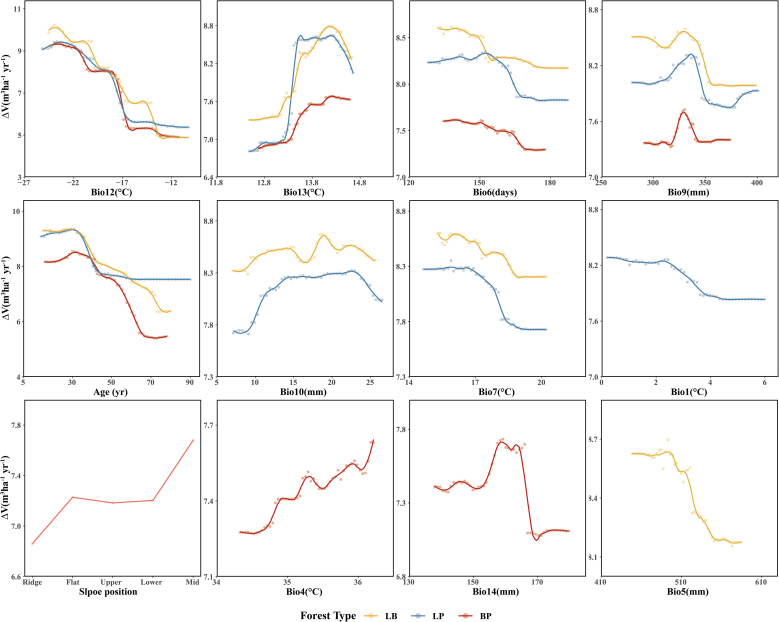
Partial dependence to the most important predictor variables of three forest types.

**Figure 9:**
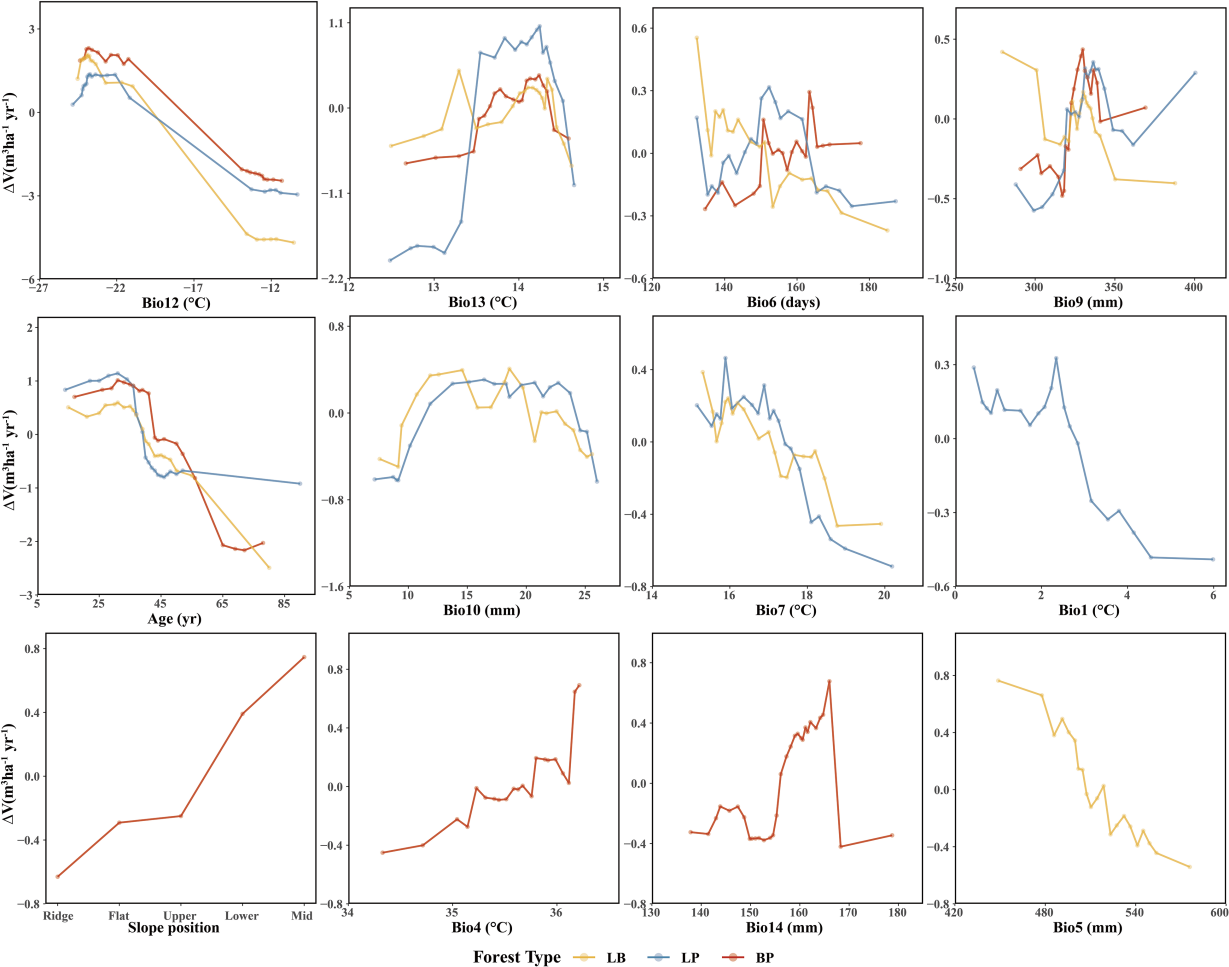
Accumulated local effects to the most important predictor variables of three forest types.

Among the three forest types, the LB stands exhibited the highest stand volume growth (Δ*V*). All three models showed a fluctuating decline in Δ*V* in response to Bio12 (**Figure 8**). The distribution of Bio12 was bimodal, concentrated in both low and high value ranges (**Figure 9**). When Bio12 was in the lower range, Δ*V* increased, while it declined at higher Bio 12 values (**Supplementary Figures S8-S10**). The response of Δ*V* to stand age showed a unimodal pattern across all forest types, peaking at approximately 30 years and gradually declining thereafter. The response of Δ*V* to Bio6 was also sensitive, with LB showing the highest Δ*V* in the high-value range of Bio6, while LP and BP exhibited the highest Δ*V* at intermediate Bio6 values. BP showed greater sensitivity to slope position, with higher Δ*V* observed at mid- and lower-slope positions.

For climatic factors, the response trends were similar across forest types, although the magnitude of variation differed. Among the precipitation-related factors, summer precipitation (Bio9), winter precipitation (Bio10), precipitation of the wettest month (Bio14), and annual precipitation (Bio5) showed similar nonlinear responses, characterized by an initial facilitation of Δ*V* followed by suppression at higher values. In contrast, temperature-related responses were more complex. The mean diurnal range (Bio13) exhibited an increase followed by a decrease in Δ*V* across all forest types. The response of LB and LP to the mean temperature of summer (Bio7) showed an overall declining trend, with higher Δ*V* at lower Bio7 values. Similarly, the response of LP to annual mean temperature (Bio1) resembled that of Bio7. In BP, the response to the monthly temperature difference (Bio4) showed a fluctuating increase, with higher Δ*V* observed at elevated Bio4 values.

## 4 Discussion

### 4.1 RF model evaluation

The 10-fold spatial cross-validation procedure demonstrated that all three random forest models (LP, BP, and LB) exhibited robust performance on the training set, confirming their effectiveness in modeling stand volume growth across different forest types. Among them, the BP model achieved the highest *R*² (0.92), followed by the LB (*R*² = 0.88) and LP (*R*² = 0.81)models. These results indicate that the RF models effectively captured the major determinants of forest growth, with particularly strong performance in the BP and LB forests. This superior performance likely reflects the inherent spatial structure and ecological complexity of these forest types. Specifically, BP forests, which are primarily natural in origin (Chen and Lou, 2019; Zhang et al., 2022a), and LB mixed forests, characterized by higher degree of complexity and environmental heterogeneity, provide richer variability for model learning and generalization. The LP model, derived mainly from homogeneous plantation stands, also demonstrated good performance, consistent with previous studies that plantations tend to exhibit strong model fit due to uniform stand structure and controlled silvicultural practices (Tian et al., 2022). However, all models exhibited reduced performance on the independent test set, which may be attributed to the intrinsic ecological complexity of forest ecosystems. These complex factors such as interspecies competition, resource partitioning, and microenvironmental heterogeneity are difficult to capture fully using standard predictor variables, which may lead to decreased predictive accuracy when applied to novel data (Liu et al., 2022).

### 4.2 Differential performance of predictors in models

Variable importance was quantified using the Percent Increase in MSE (%IncMSE), which assesses the reduction in model accuracy when a given predictor is permuted or removed (Breiman, 2001). Across all forest types, climatic variables overwhelmingly dominated model performance, with the minimum temperature of the coldest month (Bio12) emerging as the most influential factor in the LP, BP, and LB models, contributing 19.8%, 36.4%, and 28.6% to total relative importance, respectively (**Figure 4**). Stand age consistently ranked among the top eight predictors across models, reaffirming its pivotal role in regulating stand development and productivity. This finding is consistent with previous findings: Tian et al. (2022) identified stand age as the primary driver of *Larix* volume growth, while Zhang et al. (2022a) highlighted the importance of stand structure for *Betula* radial growth. Similar conclusions have been drawn in species-specific studies, where structural attributes and competition indices were found to be dominant predictors of forest productivity (Ou et al., 2019; Jevšenak and Skudnik, 2021). Beyond variable importance, Friedman’s H-statistic provided additional insights into the strength of interactions among predictors (**Figures 5**-**7**). Interaction analyses revealed that stand age exhibited the highest overall interaction effects in both LP and BP models (*H* ≈ 0.2), suggesting that developmental stage modulates the sensitivity of stands to environmental drivers. In the LP model, the interaction between stand age and canopy density (*H* = 0.16) indicated that stand structural attributes mediate the influence of climatic constraints on stand growth. In the BP model, the interaction between stand age and Bio12 (*H* = 0.07) suggested that birch growth is highly sensitive to cold stress and that temperature effects are contingent on stand maturity. In contrast, the LB model showed that Bio12 itself had the highest overall interaction strength (*H* = 0.31), with its strongest interaction observed with stand age (*H* = 0.08), implying that temperature limitations and developmental dynamics jointly regulate mixed-stand growth patterns. Integrating the %IncMSE and H-statistic results indicates that the most influential predictors—particularly Bio12 and stand age—function not only as dominant main effects but also as key interaction hubs shaping forest growth responses. These findings support previous evidence that climatic factors and stand attributes jointly regulate productivity across temperate forest ecosystems (Tian et al., 2022; Zhang et al., 2022a; Li et al., 2022; Liu et al., 2025).

### 4.3 Key factors in the stand volume growth models of three forest types

This study revealed a strong dependence of volume growth in LP (pure larch) on stand age (**Figure 8**), exhibiting a staged growth pattern characteristic of typical allometric trajectory in plantation development (Zhao et al., 2011; Qi et al., 2016). In the young (≤20 years) and middle-aged (21–40 years) stages, elevated growth rates were primarily driven by larch’s biological traits and favorable site conditions. During this period, larch accumulates trunk biomass most rapidly (Zhao et al., 2011), supported by sufficient sunlight and reduced belowground competition for nutrients (Rosenthal and Camm, 1996; Wang et al., 2017a). However, growth peaked around 30 years and subsequently declined, likely due to the effects of selective thinning. As stands entered the near-mature (41–50 years) and mature (>50 years) stages, volume growth stabilized, possibly as a result of nutrient depletion in deeper soil layers, where root expansion becomes increasingly constrained by limited nutrient availability (Wang et al., 2019). This observed partial dependence on stand age aligns with the findings of Tian et al. (2022).

LP volume growth also exhibited a threshold response to Bio12 (minimum temperature of the coldest month), likely due to premature dormancy break. Trees, adapted to low temperatures, reduces metabolic activity and enters dormancy during winter. If winter temperatures are not sufficiently low, accumulated heat may trigger early budburst, increasing vulnerability to late frost events (Gömöry et al., 2015), which in turn depletes stored carbon and nutrient reserves due to the need for re-sprouting (Rubio-Cuadrado et al., 2021). Moreover, premature budburst prior to soil thaw may induce physiological drought. Similar threshold responses were observed for Bio1, and Bio7. In the study area, temperature emerged as the primary climatic driver of LP growth, consistent with national-scale findings (Tian et al., 2022). Bio1, Bio7, and Bio13 significantly influenced growth, while other precipitation-related factors (e.g., Bio9, Bio10) had relatively minor impacts.

BP (pure birch) exhibited a similar ranking of predictor importance and response patterns to LP, reflecting convergent growth strategies under comparable environmental constraints (Gömöry et al., 2015; Oksanen, 2021). The stand volume growth of BP showed high sensitivity to slope position, particularly with more vigorous growth observed on middle and lower slopes. This phenomenon is likely related to the impact of slope position on water availability, light exposure, and soil nutrient status. Middle and lower slopes tend to accumulate more surface runoff and groundwater, providing a more stable water supply. Additionally, these slopes, particularly those with favorable aspects, generally receive greater sunlight exposure, enhancing photosynthetic activity. Lower slopes also tend to have deeper and more fertile soils due to the accumulation of organic matter and nutrients, whereas upper slopes are typically characterized by faster drainage and lower soil fertility. Consequently, BP forests on middle and lower slopes benefit from more favorable hydrothermal and edaphic conditions, leading to enhanced stand volume growth.

In LB (larch-birch mixed forest), volume growth was consistently higher than that in pure stands (**Figure 8**), suggesting that moderate interspecific competition enhances mutual facilitation, in line with findings by Zhang et al. (2022b). The diurnal temperature range (Bio13) also exerted a significant effect: moderate increases enhanced energy accumulation and growth (Fu et al., 2016), whereas excessive daytime temperatures increased transpiration rates and water loss, leading to growth suppression (Sadok et al., 2021). The increased relative importance of Bio13 suggests that interspecific competition amplifies sensitivity to thermal variability, consistent with the findings of Forrester (Forrester, 2015). In both LP and BP pure forest types, the response to the frost-free period (Bio6) shows an adaptation to moderate climatic conditions. In these forests, stand growth is more vigorous under moderate frost-free periods, suggesting that such conditions provide an optimal balance between temperature, moisture availability, and physiological activity. This moderate duration likely supports sustained metabolic processes and long-term growth stability in these forest types. In contrast, LB mixed forests exhibited the highest growth under shorter frost-free periods, implying greater adaptability to cooler or more variable climatic conditions. This pattern may reflect reduced interspecific competition and enhanced resource-use efficiency in mixed stands, enabling them to maintain higher productivity even under shorter growing seasons.

### 4.4 Study limitations and recommendations

The RF models demonstrated strong predictive performance across all three stand types, owing to the algorithm’s robust capability to interpret high-dimensional datasets. However, the representation of stand structure in this study was limited, relying solely on stand age and canopy density as structural predictors of volume growth. This limitation may have affected the model’s generalization capacity by restricting its ability to capture the underlying structural complexity of forest ecosystems. While the lack of detailed structural variables likely contributed to this limitation, other sources of uncertainty—such as potential bias in volume equations and the exclusion of disturbance history—should also be considered when interpreting the model outputs. Moreover, the exclusive reliance on the Random Forest (RF) model, without comparison to alternative approaches such as linear mixed-effects models (LMEM), generalized additive models (GAM), or gradient boosting methods (GBM), constitutes an limitation of this study. Although RF is well suited for analyzing complex and high-dimensional ecological data, future research should incorporate multiple modeling frameworks to benchmark performance and evaluate the generalizability of predictions across diverse forest types and climatic conditions. Such comparative analyses would provide a more comprehensive assessment of model suitability and strengthen confidence in the resulting predictions.

Future research should also incorporate a broader range of stand structural parameters, including interspecific competition indices, tree size distributions, and niche overlap metrics, to better represent ecosystem complexity and improve model performance. The high relative importance of Bio12 highlights the significant stress imposed by extreme climatic events and emphasizes the necessity of incorporating disturbance-related variables into future models’ frameworks. Notably, mixed stands exhibited higher productivity than pure stands, indicating that species diversity enhances stand stability under climate variability. Based on these findings, forest management practices in the mountainous region of northern Hebei should prioritize optimizing stand structure and mitigating the effects of climate change on forest productivity. Recommended strategies include the underplanting of complementary tree species to improve the structural and functional diversity of pure stands, as well as the implementation of adaptive management practices in near-mature and mature forests to promote continued volume growth.

## 5 Conclusions

This study employed Random Forest (RF) models to predict the volume growth of larch (LP), birch (BP), and mixed larch–birch (LB) forests in northern China, using key climatic and environmental variables. The models demonstrated strong predictive performance, with the BP model achieving the highest explanatory power (R² = 0.92). Among the predictors, the minimum temperature of the coldest month (Bio12) emerged as the most influential factor across all forest types, underscoring the dominant role of temperature in regulating forest productivity. Stand age also exhibited a strong effect on volume growth and showed pronounced interactions with other predictors, particularly in the LP and BP models. The analysis further revealed distinct growth-regulating mechanisms among forest types: temperature-related factors and stand age were critical drivers for LP and BP stands, whereas the enhanced growth observed in LB mixed forests was largely attributed to species diversity and interspecific facilitation. These results suggest that mixed forests possess greater resilience to climatic variability and disturbance, leading to higher productivity than pure stands. From a management perspective, optimizing forest structure, promoting species diversity, and implementing adaptive management practices should be prioritized to enhance forest stability and productivity in the mountainous regions of northern Hebei. Future research should integrate additional structural descriptors, such as tree size distribution, interspecific competition indices, and disturbance history, to refine model accuracy and deepen understanding of forest growth dynamics under changing climatic conditions.

## Data Availability

The datasets presented in this study can be found in online repositories. The names of the repository/repositories and accession number(s) can be found in the article/**Supplementary Material**.
