# Supplementary figures and images for: Using a random forest model to predict volume growth of larch, birch, and their mixed forests in northern China
Source: Front Plant Sci. 2025 Dec 2;16:1682940. doi: 10.3389/fpls.2025.1682940 (PMC12705547; doi:10.3389/fpls.2025.1682940)

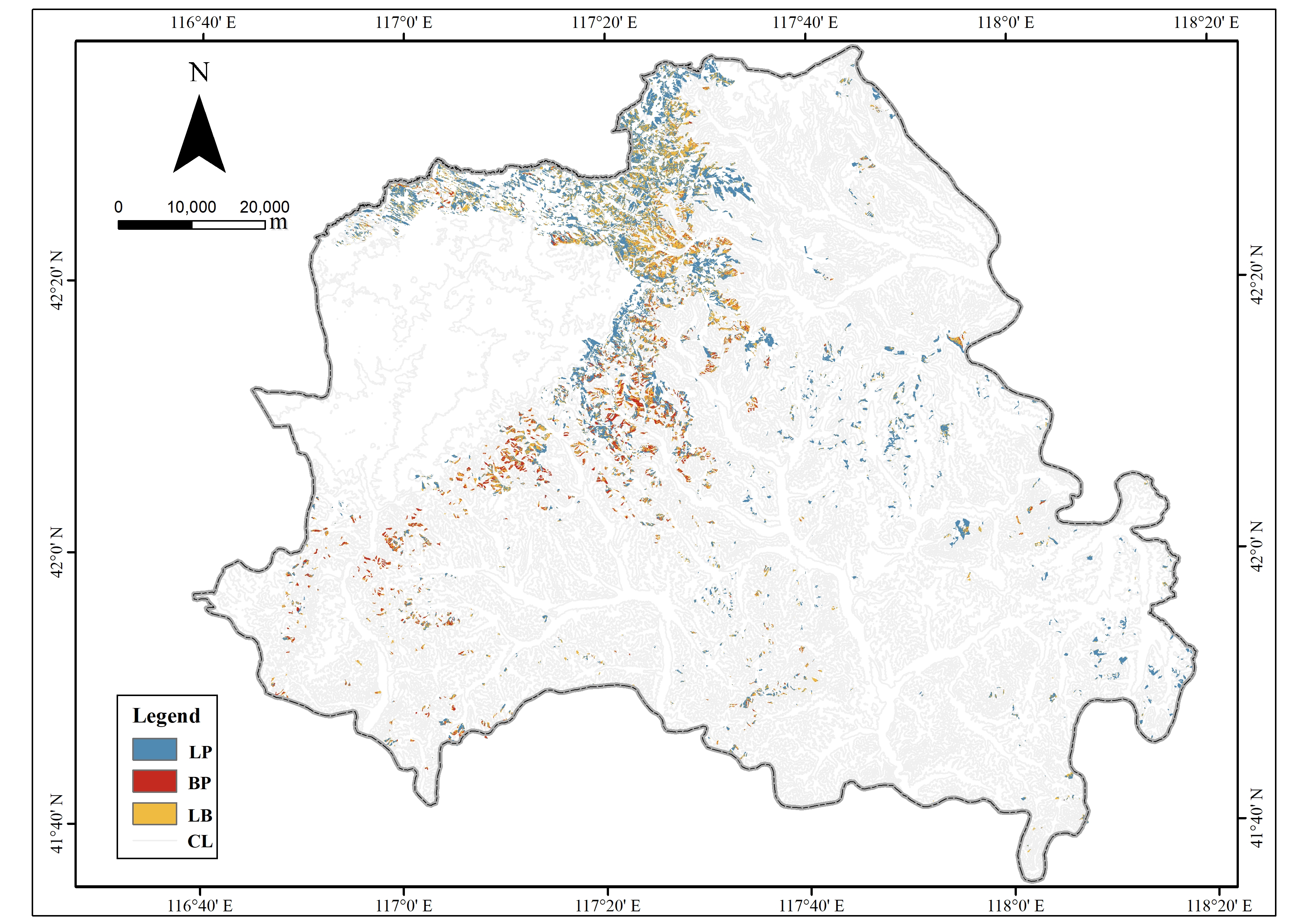

Supplement: Supplementary file 1 [file Image1.jpeg]

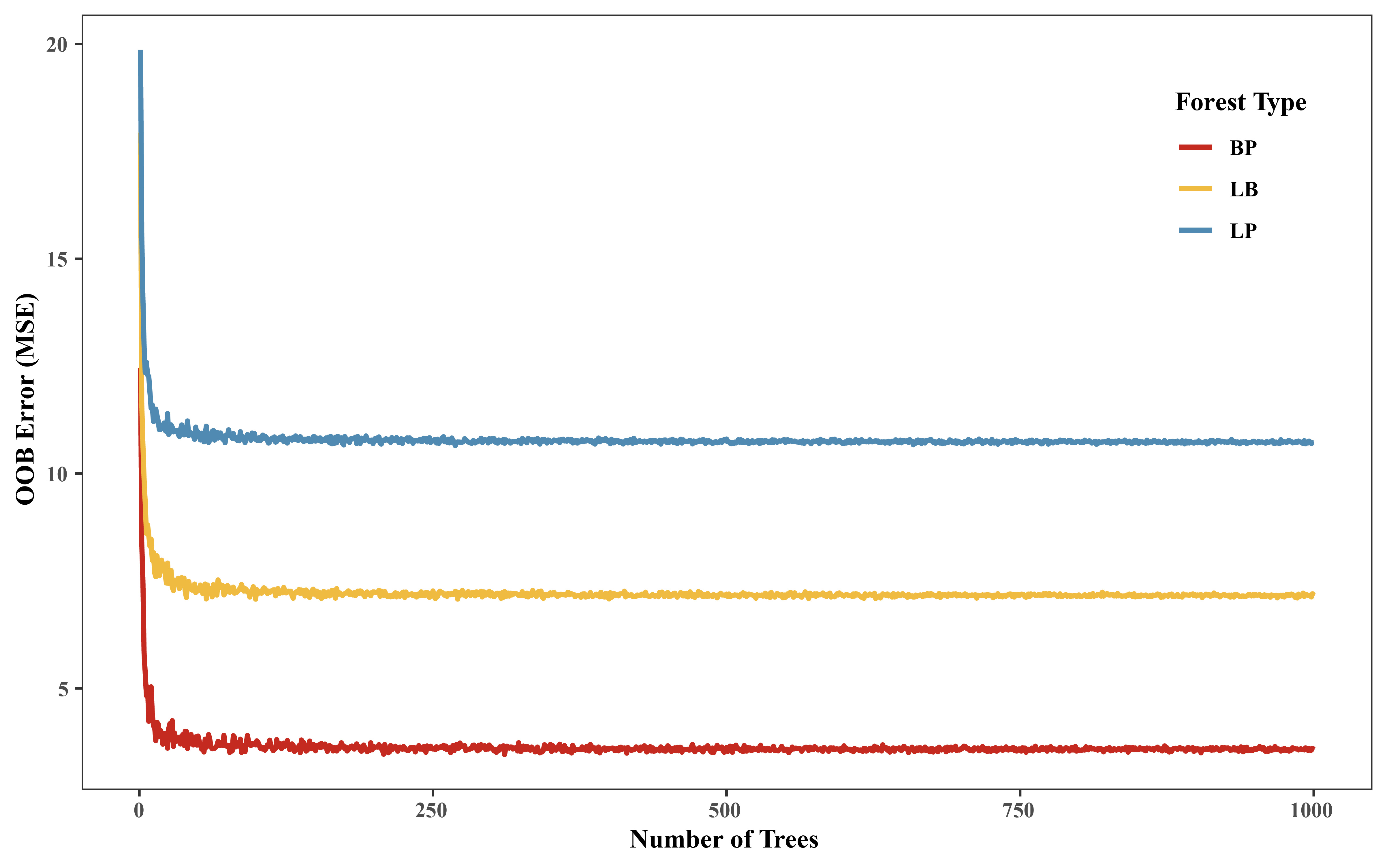

Supplement: Supplementary file 2 [file Image2.jpeg]

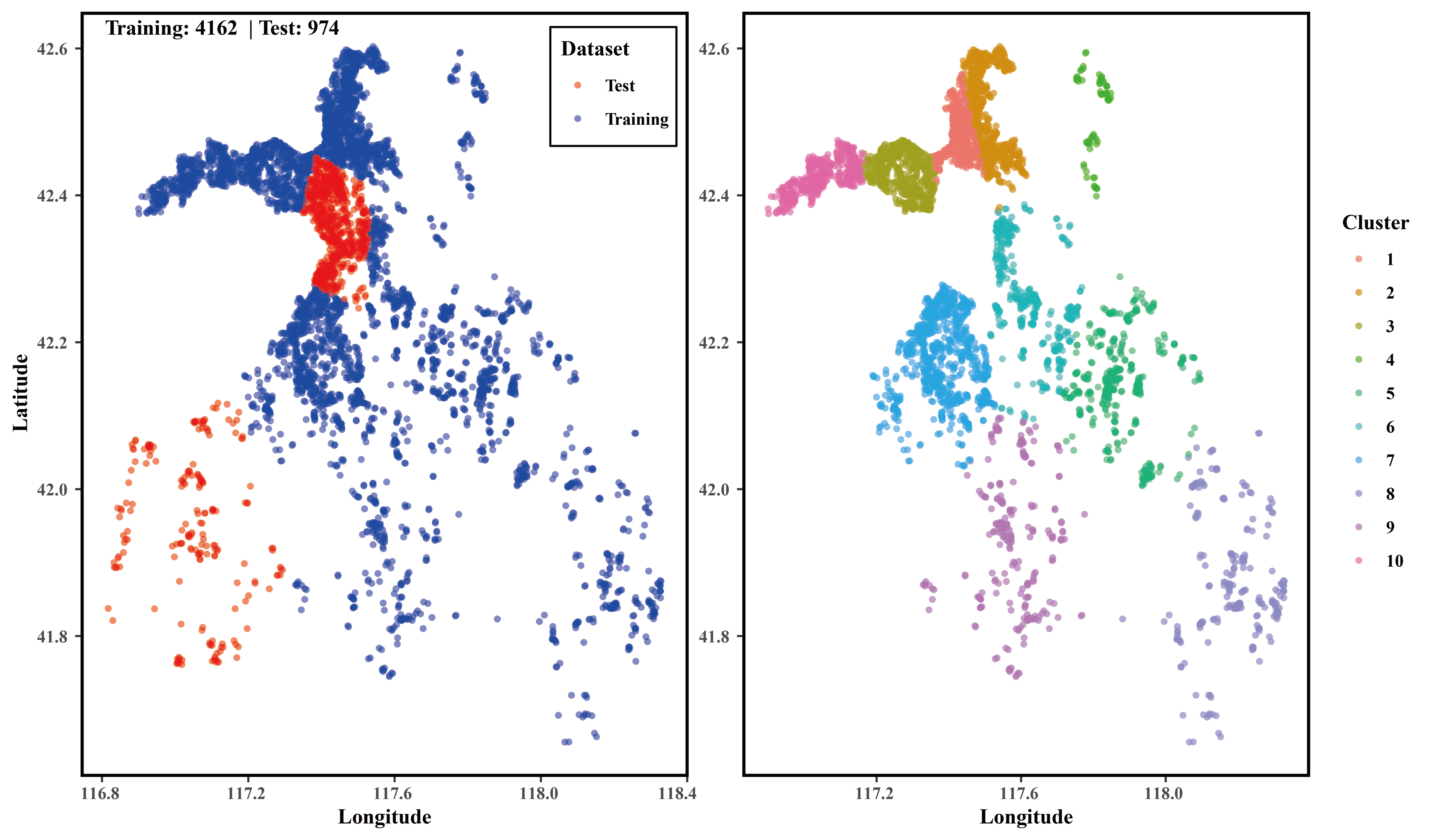

Supplement: Supplementary file 3 [file Image3.jpeg]

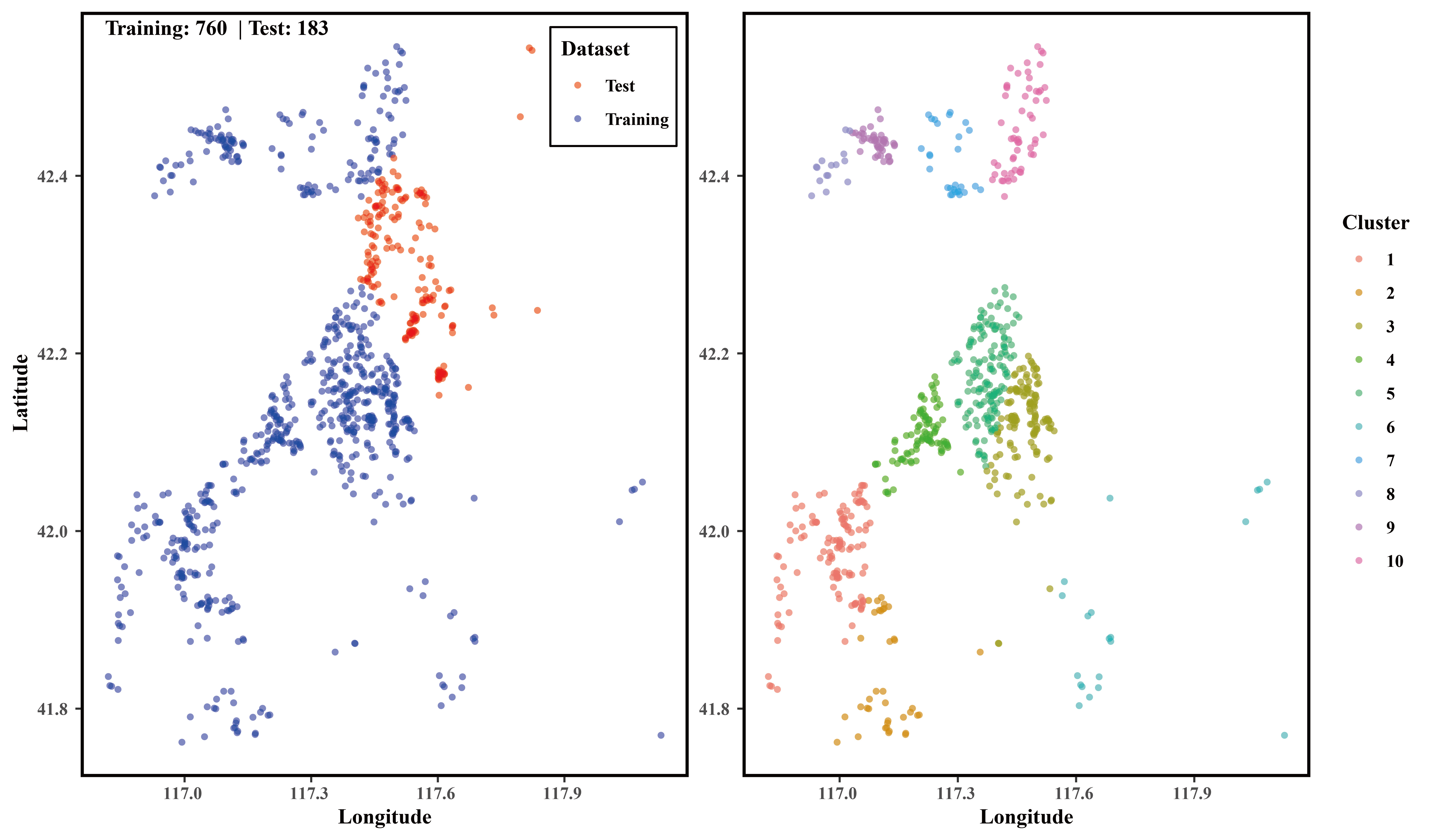

Supplement: Supplementary file 4 [file Image4.jpeg]

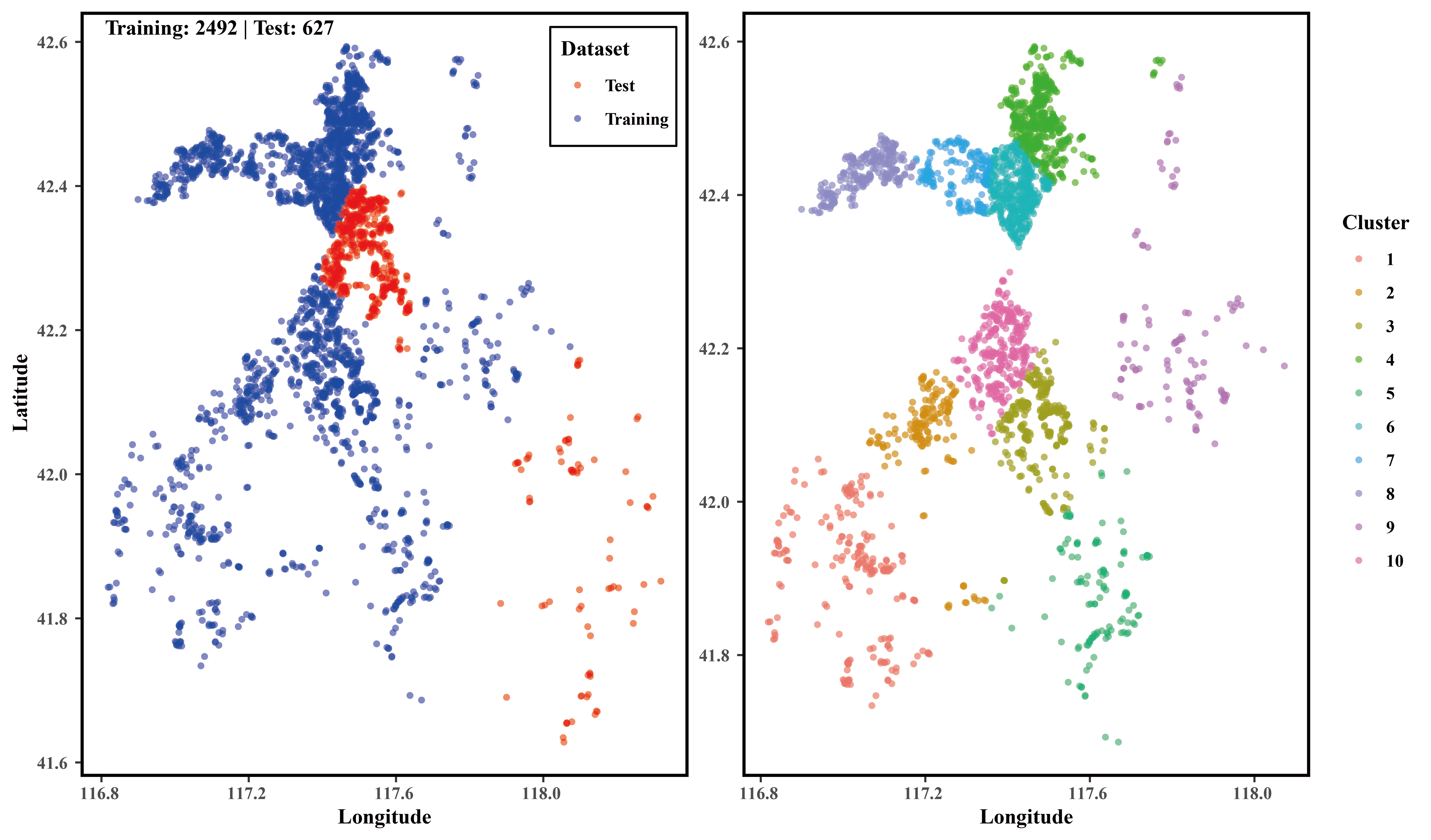

Supplement: Supplementary file 5 [file Image5.jpeg]

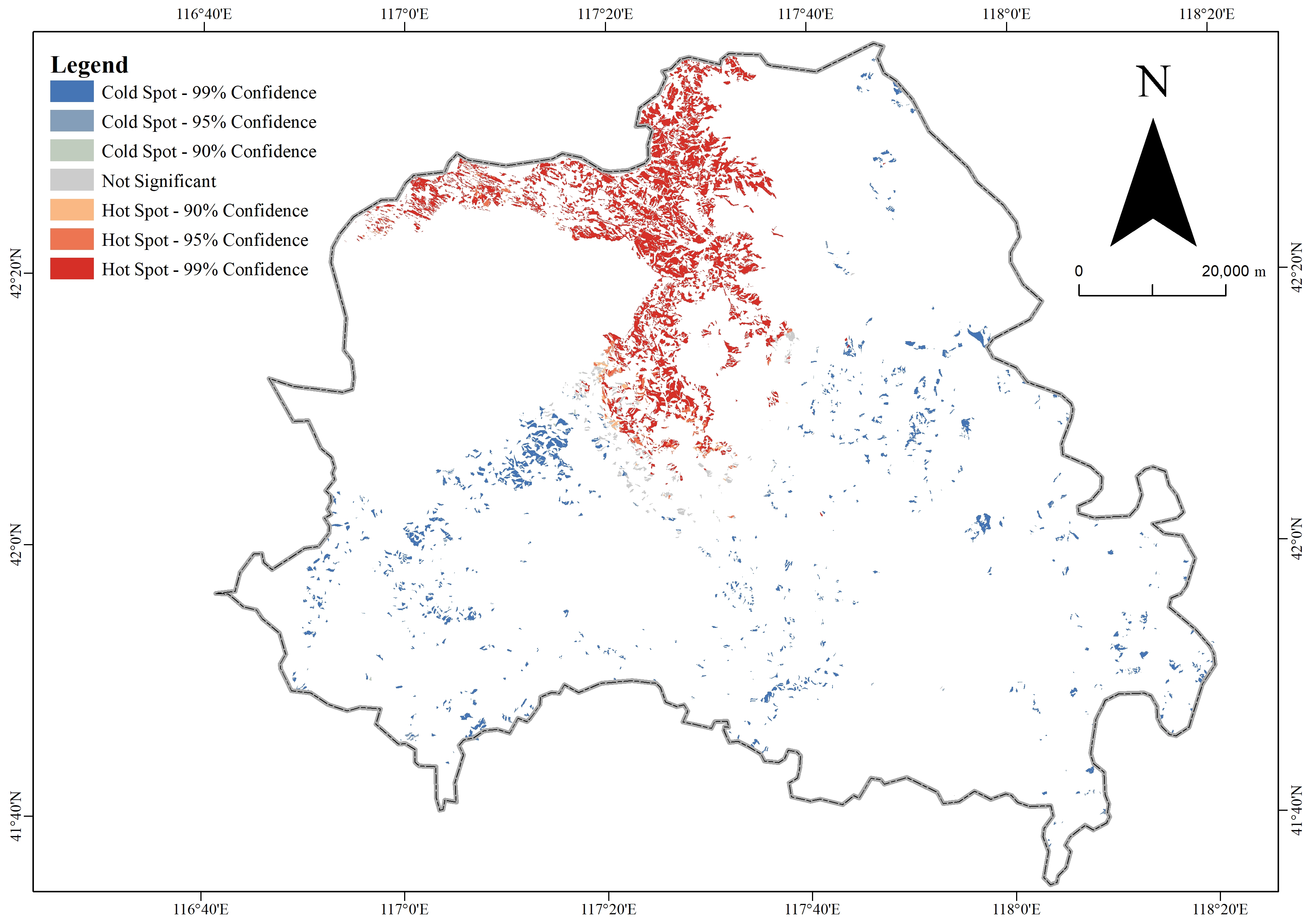

Supplement: Supplementary file 6 [file Image6.jpeg]

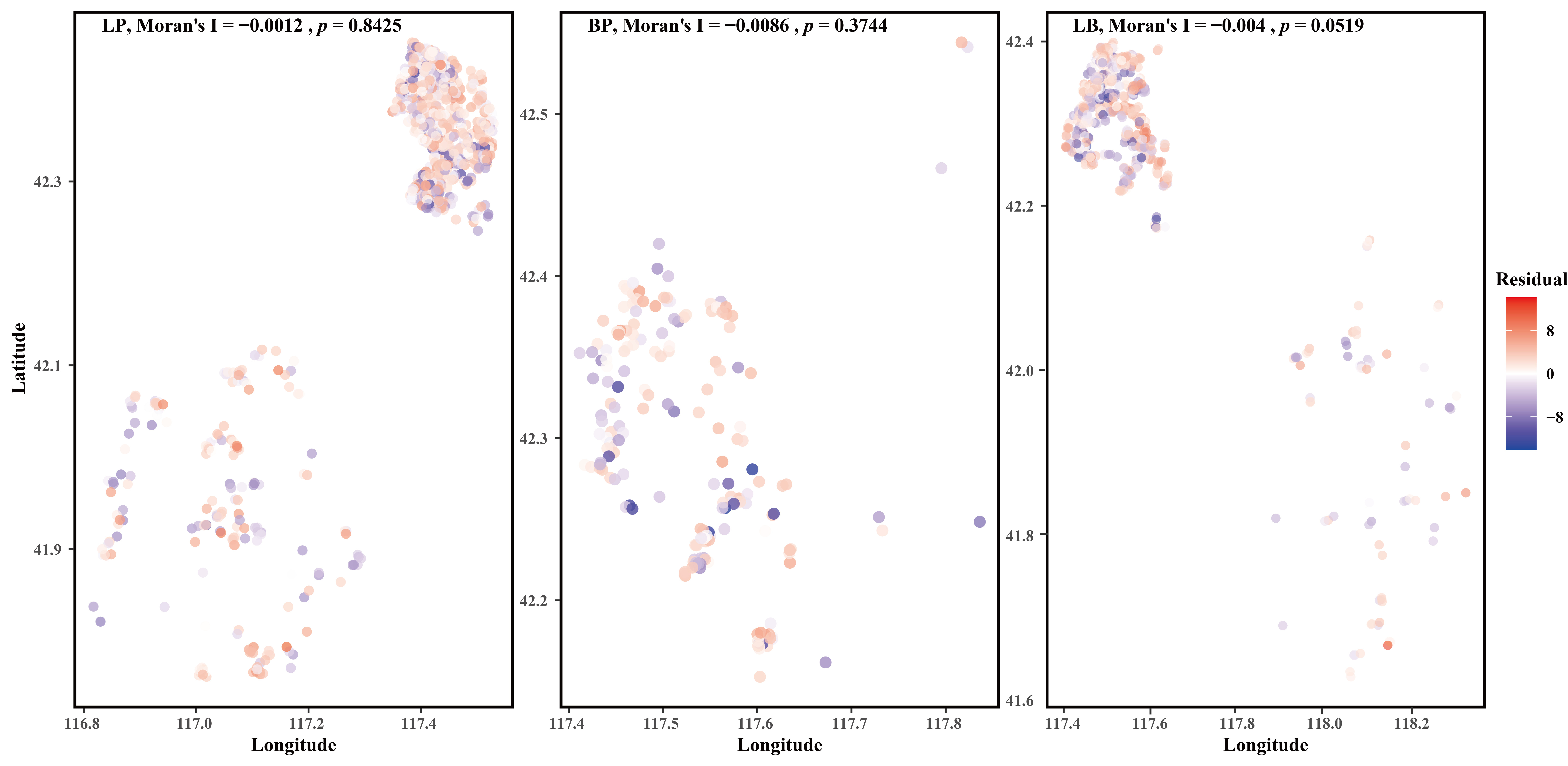

Supplement: Supplementary file 7 [file Image7.jpeg]

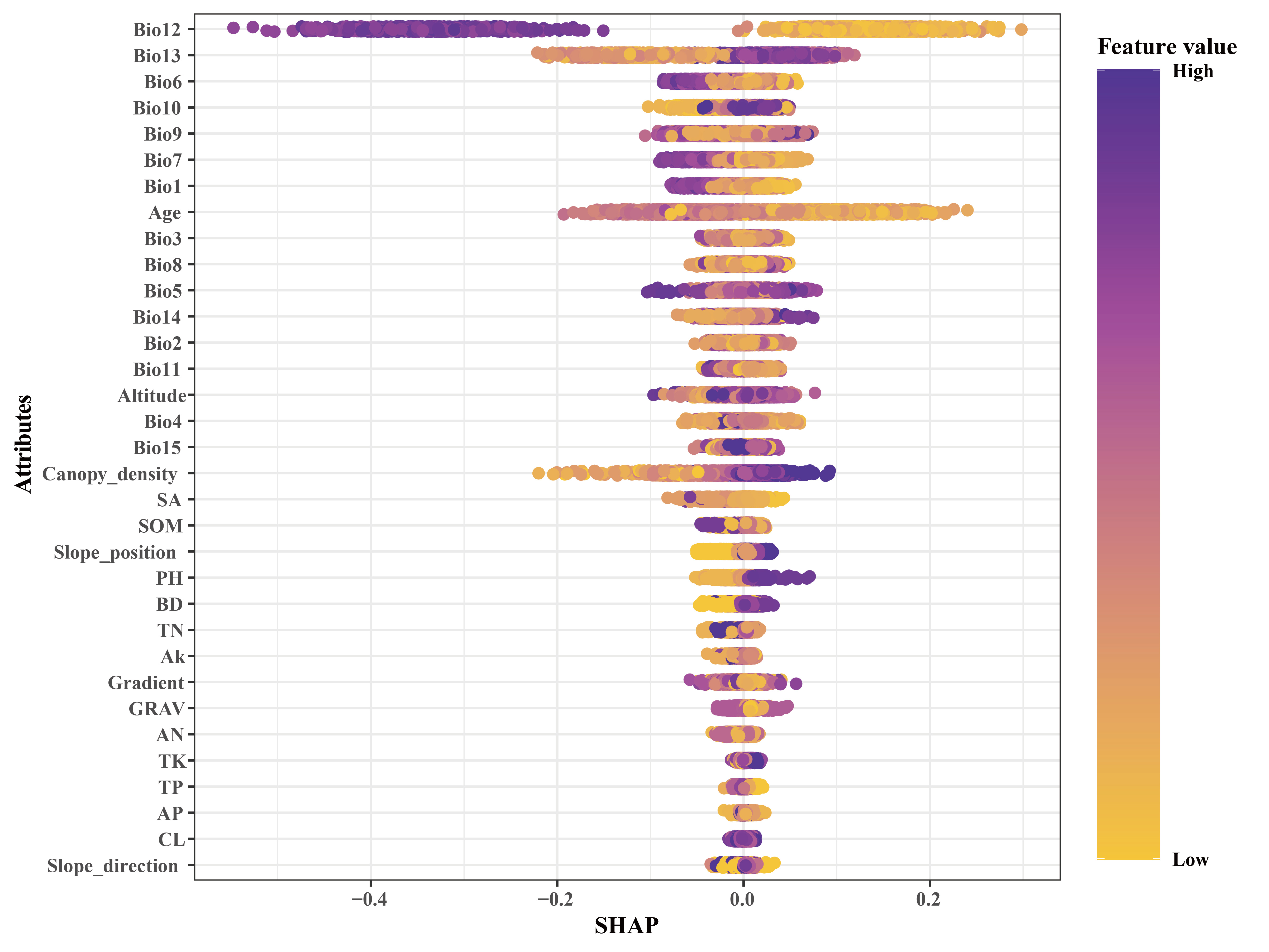

Supplement: Supplementary file 8 [file Image8.jpeg]

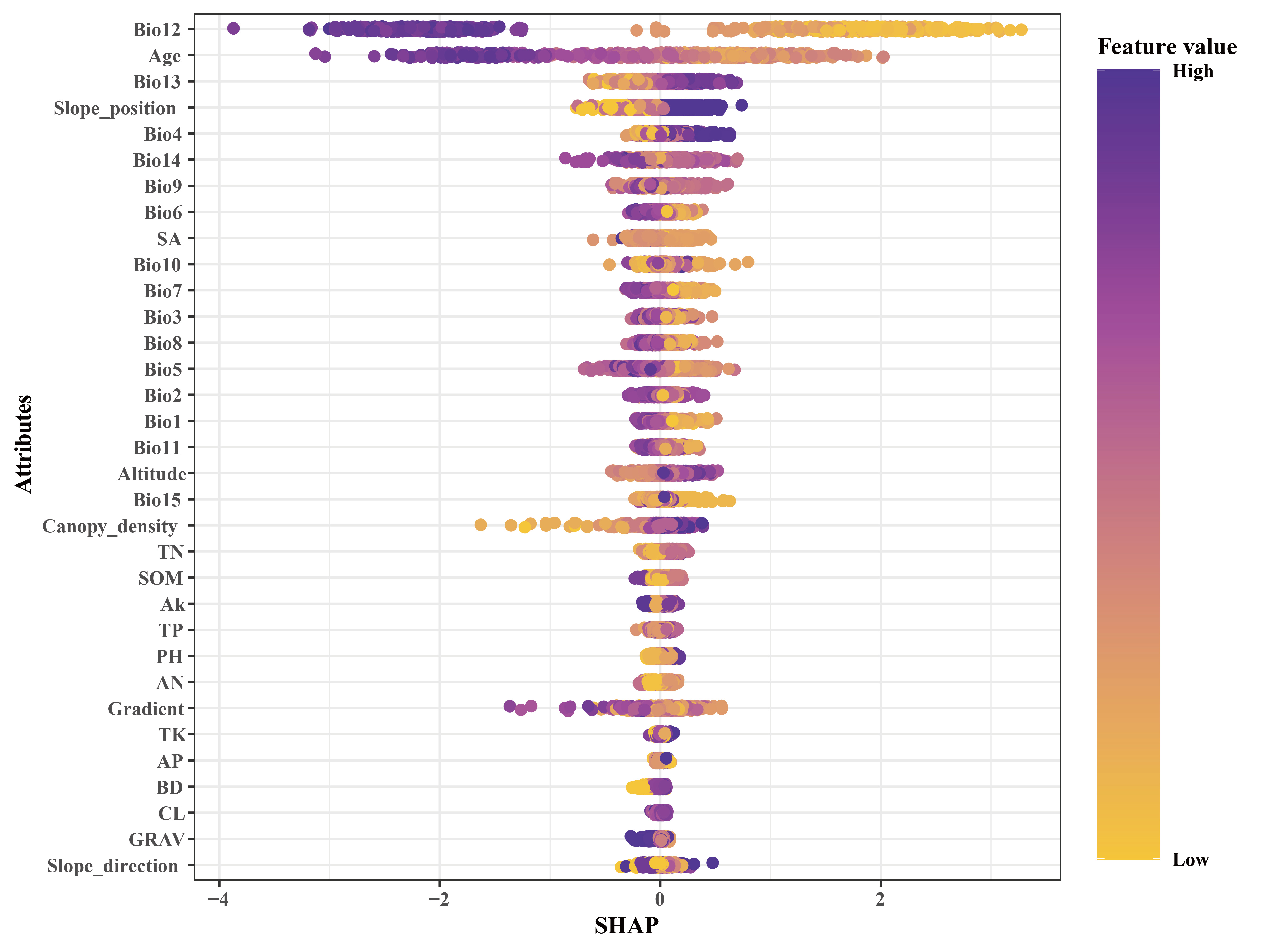

Supplement: Supplementary file 9 [file Image9.jpeg]

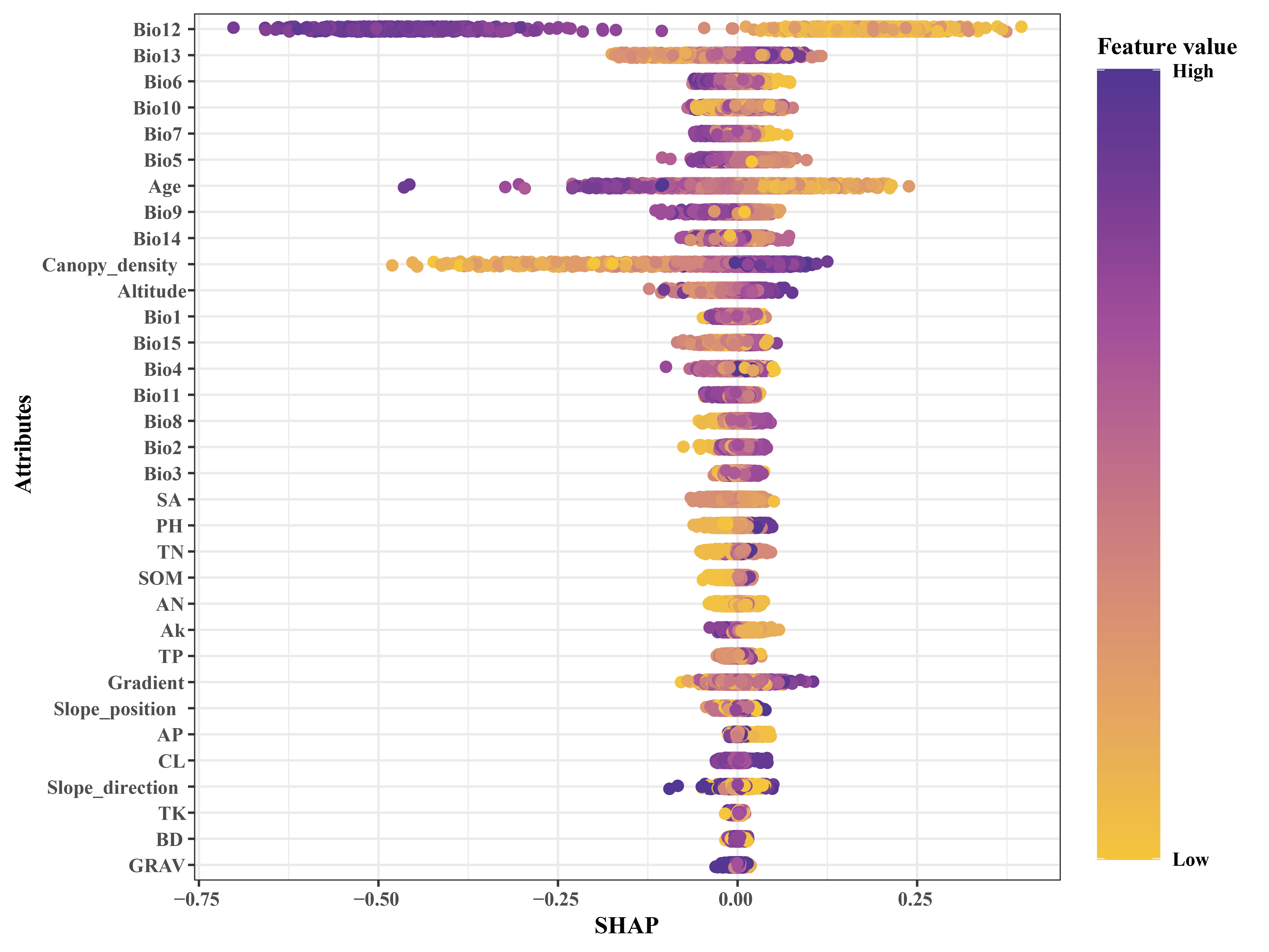

Supplement: Supplementary file 10 [file Image10.jpeg]
