# Supplementary material for: Using a random forest model to predict volume growth of larch, birch, and their mixed forests in northern China
Source: Front Plant Sci. 2025 Dec 2;16:1682940. doi: 10.3389/fpls.2025.1682940 (PMC12705547; doi:10.3389/fpls.2025.1682940)
Supplement: Supplementary file 11 [file Table1.docx]

Supplementary Material

# Supplementary Table

Table S1. Classification accuracy assessment.

| Stand type | LP | BP | LB | Row total | PA (%) |
| --- | --- | --- | --- | --- | --- |
| LP | 55 | 1 | 1 | 57 | 96.49 |
| BP | 1 | 53 | 3 | 57 | 92.98 |
| LB | 4 | 6 | 56 | 66 | 84.85 |
| Column total | 60 | 60 | 60 | 180 |  |
| UA (%) | 91.67 | 88.33 | 93.33 |  |  |
| Overall classification accuracy = 91.11%, Kappa Coefficient = 86.67% | | | | | |

Note: LP, *Larix principis-rupprechtii* pure forest; BP, *Betula platyphylla* pure forest; LB, *L. principis-rupprechtii–B. platyphylla* mixed forest; PA, Producer’s Accuracy, UA, User’s Accuracy.
