# Supplementary material for: Using a random forest model to predict volume growth of larch, birch, and their mixed forests in northern China
Source: Front Plant Sci. 2025 Dec 2;16:1682940. doi: 10.3389/fpls.2025.1682940 (PMC12705547; doi:10.3389/fpls.2025.1682940)
Supplement: Supplementary file 12 [file Table2.docx]

Supplementary Material

# Supplementary Table

Table S2. Applicability Scope of Single-Tree Volume Equations

| Equation | Applicable Regions | D Range | Key Performance Metrics |
| --- | --- | --- | --- |
| Equation (1) | Beijing, Tianjin, Hebei, Shanxi, and Inner Mongolia (excluding its eastern part). | D ≥ 5 cm | R² = 0.99, MPE = 2.41% |
| Equation (2) | Beijing, Tianjin, Hebei, Shanxi, Inner Mongolia (excluding its eastern part), Shaanxi, Gansu, Qinghai, Ningxia, and Xinjiang. | D ≥ 5 cm | R² = 0.93, MPE = 5.67% |

Note: The eastern part of Inner Mongolia refers to Hulunbuir City and Xing'an League.
